# Prevalence of cervicovaginal human papillomavirus infection and genotype distribution in Shanghai, China

**DOI:** 10.1186/s12985-022-01879-y

**Published:** 2022-09-12

**Authors:** Xiaoxiao Li, Fenfen Xiang, Junhua Dai, Tao Zhang, Zixi Chen, Mengzhe Zhang, Rong Wu, Xiangdong Kang

**Affiliations:** Laboratory Medicine Department, Putuo Hospital, Shanghai University of Traditional Chinese Medicine, 164 Lanxi Road, Shanghai, 200062 People’s Republic of China

**Keywords:** Human papillomavirus, Prevalence, Genotype, Vaccine, Cervical cancer

## Abstract

**Background:**

The evaluation of human papillomavirus (HPV) prevalence rate dynamics and genotype distribution could support the adoption of more targeted prevention and treatment of cervical cancer. We aimed to assess the infection status and genotype characteristics of HPV among gynecological outpatients in Shanghai, China.

**Methods:**

Clinical specimens were collected from patients attending gynaecological department of the Putuo Hospital, Shanghai University of Traditional Chinese Medicine, between January 2015 and December 2019. The cervicovaginal infection of 17 high-risk genotypes and 10 low-risk genotypes were analyzed by Luminex-based multiple assays.

**Results:**

The overall HPV infection rate was 18.81% (95% CI 18.31–19.30%) in Shanghai city, with high-risk, low-risk and mixed high- and low-risk HPV prevalence being 11.65% (95% CI 11.24–12.06%), 4.19% (95% CI 3.94–4.44%) and 2.96% (95% CI 2.74–3.17%), respectively. The five most prevalent high-risk genotypes were HPV-52 (2.95%), HPV-16 (2.34%), HPV-58 (2.07%), HPV-53 (1.67%) and HPV-39 (1.36%). The most common low-risk genotype was HPV-61 (1.52%), followed by HPV-6 (1.29%) and HPV-81 (1.19%). Moreover, the coverage of HPV genotype by nonavalent vaccine was 10.42%, and non-vaccine-covered high-risk genotype was 7.70%. The 15–24 years age group demonstrated the highest HPV prevalence (43.14%), and significant differences were observed among different age groups (*P* < 0.001).

**Conclusions:**

This study revealed the HPV prevalence and genotype distribution among women in Shanghai city, which could serve as guidance for HPV vaccination and preventative strategies against cervical cancer in this area.

**Supplementary Information:**

The online version contains supplementary material available at 10.1186/s12985-022-01879-y.

## Introduction

Human papillomavirus (HPV) is one of most commonly encountered sexually transmitted infection, which mainly causes cervical cancer and other cancers (vaginal, vulvar, anal, penile and oropharyngeal cancer) [1]. More than 200 HPV genotypes have been identified, and they can be classified into high-risk (HR) and low-risk HPV (LR) genotypes based on their carcinogenicity. It has been demonstrated that persistent infection with HR HPV genotype including HPV16/18/31/33/35/39/45/52/58/59 is a major cause of cervical precancerous lesions and cervical cancer [2, 3], while LR-HPVgenotypes such as HPV6/11 are associated with condyloma acuminatum or hyperplastic lesions [4]. Approximately 99% cervical cancers were associated with HPV infection worldwide [5], and there are over 130,000 women suffered from cervical cancer per year in China [6]. Therefore, HPV detection screening is of great importance to reduce the burden of cervical cancer and other HPV-related diseases.

Currently, three licensed HPV vaccines are available, including bivalent (HPV-16 and -18), quadrivalent (HPV-6, -11, -16 and -18) and nonavalent vaccines (HPV-6, -11,-16, -18, -31, -33, -45, -52 and -58) in mainland China. They were launched and approved for use by China Food and Drug Administration (CFDA) in 2016, 2017 and 2018, respectively [7]. However, all these commercially vaccines only provide protection against a few genotypes, which were based on epidemiological data from western countries [8]. Furthermore, the HPV infection rate and genotype distribution vary by countries and regions [9, 10]. For instance, HPV-31 and HPV-33 are more frequent in Europe and America, whereas HPV-52 and -58 are more prevalent in Asia and HPV-35 and -45 in Africa [4]. Recent a meta-analysis indicated the most prevalent genotypes were HPV-16, -52 and -58, followed by HPV-18, -31, -33 and -35 in women with normal uterine cervix in different regions of China [11]. Thus, acquiring updates on prevalence and distribution of HPV genotypes among different areas will provide crucial information for decision on HPV vaccination program and development of new vaccine in China.

Shanghai have 16 districts with over 20 million population in its area. Previous studies have investigated the prevalence and genotype distribution of HPV in Zhoupu [12], Minghang [13] and Songjiang district [14] of Shanghai China. However, there is still limited information on the distribution of HPV infection in Shanghai, China. The primary objective of this study was to investigate the prevalence and genotype distribution of HPV infection among women attending gynecology clinics in Putuo district of Shanghai and to further evaluate the infection patterns in terms of age groups and geographical areas.

## Materials and methods

### Study participants

From Janauary 2015 to December 2019, women who attended at the Shanghai Putuo Hospital and Liqun Hospital and received HPV DNA genotyping test were included in this restrospective and cross-sectional study. Inclusion criteria for individual were as follows: (1) was ≥ 15 years old; (2) was living in Shanghai city; (3) was first time to receive the test and did not have any treatment; (4) not pregnant and had sexual activity. Finally, a total of 23,866 women with results of genotype-specific HPV were enrolled in this study. This study was approved by the ethics committees of Putuo Hospital, Shanghai University of Traditional Chinese Medicine (PTEC-A-2020-24-1), and the written informed consent was obtained from all the participants at each clinic visit.

### Specimen collection

Cervicovaginal cell samples were collected from each participant by professional gynecologists using plastic brushes (Tellgen Life Science, Shanghai, China). The brushes were placed into sterile tubes containing 3 ml of cell preservation solution (Tellgen Life Science, Shanghai, China) and stored at 4 °C, and finally trasported to our clinical lab within one week for HPV DNA genotype testing.

### DNA extraction and HPV genotyping

HPV DNA extraction was performed using a domestic commercial available viral DNA extraction kit (Tellgen Life Science, Shanghai, China) according to manufacturers, procedure. HPV detection and genotyping were conducted using Tellgenplex™ HPV DNA Test (Tellgen Life Science, Shanghai, China). The Test is a suspension bead array method that involves PCR, hybridization onto a bead using amplified PCR products and digital singnal processing [8]. In brief, 5 μL of the extracted DNA was used in the 15 μL PCR master mix reaction solution, followed by hybridization with oligonucleotide probes at 95 °C for 5 min, and 48 °C for 30 min.The hybridization product was stained with streptavidin-R-phycoerythrin, and analyzed by Luminex 200. The HPV assay kit detects 27 genotypes, including 17 high-risk (HR) HPV genotypes (HPV-16, -18, -26, -31, -33, -35, -39, -45, -51, -52, -53, -56, -58, -59, -66, -68, and -82) and 10 low-risk (LR) HPV genotypes (HPV-6, -11, -40, -42, -43, -44, -55, -61, -81, and -83).

### HPV genotype categories

Beides overall HPV, type-specific, defined HR, LR and mixed HPV prevalence, we also analyzed the prevalence of several different groupings of HPV genotypes including: (1) categories based on licensed vaccine-preventable HPV genotypes: bivalent (2v) (HPV-16/18), quadrivalent (4v) (HPV-6/11/16/18), nonavalent (9v) (HPV-6/11/16/18/31/33/45/52/58) and non-vaccine HR-HPV (HPV-26/35/39/51/53/56/59/66/68/82); (2) categories grouped according infection feature: single and multiple types infection. (3) responsive classification implicated in oncogenic degree (HPV-16/18/31/33/35/39/45/51/52/56/58/59/68). In addition, The participants were divided into six groups by age (≤ 24, 25–34, 35–44, 45–54, 55–64, ≥ 65), and the prevalence of HPV infection in those age groups was calculated. Moreover, the potential impact of current vaccines in different age groups was also evaluated.

### Statistical analysis

Excel (version 2010), SPSS software (version 22.0) were used for data processing and analysis. Bubble plots were created with ggplot2 and reshape2 packges, and heatmap plot was conducted with pheatmap package in R (version 4.1.2). The 95% confidence interval (CI) for HPV prevalence was estimated. Considering the impact of 4v and 9v vaccines, McNemar exact test and multiple chi-square test were used to compare the significant difference between two paired percentages and prevalence among different groups, respectively. The prevalence of 17 HR-HPV infection was summarized according geographical division of China based on epidemiological studies published from Janauary 2015 to August 2021. Difference in 17 HR-HPV genotypes distribution were visualized by Non-metric Multi-Dimensional Scaling (NMDS) using PAST software [15]. The linear-by linear association and gamma value were used to evaluate the trend in HPV prevalence over the five years, and *P* value < 0.05 was considered statistically significant for all analyses.

## Results

### Overall and type-specific HPV prevalence

There were 23,866 women aged ≥ 15 years included in the study. The overall prevalence of HPV infection was 18.81% (4489 cases, 95% CI 18.31–19.30%). Single and multiple HPV infection accounted for 13.46% (95% CI 13.03–13.89%) and 5.35% (95% CI 5.06–5.63%) of all the participants. In the multiple infection, double HPV genotypes infection was the most common feature (3.62%). The prevalence rates for HR-HPV, LR-HPV and mix-risk HPV were 11.65% (95% CI 11.24–12.06%), 4.19% (95% CI 3.94–4.44%) and 2.96% (95% CI 2.74–3.17%), respectively. Among all of the HR-HPV women, single HR-HPV positive rate was 9.67%, which was significantly higher than that of multiple HR-HPV infection (1.99%, *P* < 0.001). A similar results was also observed for LR-HPV infection (single HR-HPV: 3.80% vs multiple LR-HPV: 0.40%, *P* < 0.001). A downward trend of the HPV prevalence was observed based on year (*P* < 0.001; Tables 1, 2).

**Table 1:** The prevalence of HPV infection by different characteristics from 2015 to 2019

	Positive cases	Prevalence (%)	95% CI for all samples	2015 (n = 252)	2016 (n = 1900)	2017 (n = 2728)	2018 (n = 5907)	2019 (n = 13,079)
*Infection feature*								
1	3213	13.46	13.03–13.89	80 (31.75%)	314 (16.53%)	413 (15.14%)	733 (12.41%)	1673 (12.79%)
2	863	3.62	3.37–3.85	34 (13.49%)	106 (5.58%)	109 (4.00%)	196 (3.32%)	418 (3.20%)
3	248	1.04	0.91–1.16	13 (5.16%)	24 (1.26%)	38 (1.39%)	49 (0.83%)	124 (0.95%)
4	97	0.41	0.32–0.48	7 (2.78%)	14 (0.74%)	12 (0.44%)	18 (0.30%)	46 (0.35%)
5	40	0.17	0.11–0.21	3 (1.19%)	7 (0.37%)	7 (0.26%)	7 (0.12%)	16 (0.12%)
6	16	0.07	0.03–0.10	3 (1.19%)	3 (0.16%)	2 (0.07%)	3 (0.05%)	5 (0.04%)
≥ 2	1276	5.35	5.06–5.63	62 (24.60%)	158 (8.32%)	169 (6.20%)	276 (4.67%)	611 (4.67%)
*Type of infection*								
Single HR	2307	9.67	9.29–10.04	58 (23.02%)	225 (11.84%)	301 (11.03%)	555 (9.40%)	1168 (8.93%)
Single LR	906	3.80	3.55–4.03	22 (8.73%)	89 (4.68%)	112 (4.11%)	178 (3.01%)	505 (3.86%)
HR + HR	474	1.99	1.80–2.16	15 (5.95%)	58 (3.05%)	69 (2.53%)	102 (1.73%)	230 (1.76%)
HR + LR	707	2.96	2.74–3.17	42 (16.67%)	87 (4.58%)	85 (3.12%)	154 (2.61%)	339 (2.59%)
LR + LR	95	0.40	0.31–0.47	5 (1.98%)	13 (0.68%)	15 (0.55%)	20 (0.34%)	42 (0.32%)
*Vaccine genotype*								
2v	776	3.25	3.03–3.48	23 (9.13%)	89 (4.68%)	106 (3.89%)	195 (3.30%)	363 (2.78%)
4v	1213	5.08	4.80–5.36	57 (22.62%)	165 (8.68%)	168 (6.16%)	269 (4.55%)	554 (4.24%)
9v	2487	10.42	10.03–10.81	103 (40.87%)	280 (14.74%)	329 (12.06%)	600 (10.16%)	1175 (8.98%)
Non-vaccine HR-HPV	1837	7.70	7.36–8.03	56 (22.22%)	201 (10.58%)	235 (8.61%)	430 (7.28%)	915 (7.00%)

**Table 2:** Overall and type-specific prevalence of HPV infection, 2015–2019

HPV genotype	Positive cases	Percentage for all sample (%)	95% CI for all samples (%)	2015 (n = 252)	2016 (n = 1900)	2017 (n = 2728)	2018 (n = 5907)	2019 (n = 13,079)	χ^2^	*P*	gamma value
Any HPV	4489	18.81	(18.31–19.30)	142 (56.35%)	472 (24.84%)	582 (21.33%)	1009 (17.08%)	2284 (17.46%)	155.025	< 0.001	− 0.125
HR HPV	2781	11.65	(11.24–12.06)	73 (28.97%)	283 (14.89%)	370 (13.56%)	657 (11.12%)	1398 (10.69%)	74.789	< 0.001	− 0.115
HPV-16	559	2.34	(2.15–2.53)	17 (6.75%)	62 (3.26%)	87 (3.19%)	139 (2.35%)	254 (1.94%)	37.216	< 0.001	− 0.185
HPV-18	239	1.00	(0.87–1.12)	6 (2.38%)	33 (1.74%)	23 (0.84%)	65 (1.10%)	112 (0.86%)	11.689	0.001	− 0.152
HPV-26	13	0.05	(0.02–0.08)	0 (0.0%)	3 (0.16%)	1 (0.04%)	3 (0.05%)	6 (0.05%)	1.308	0.253	− 0.200
HPV-31	82	0.34	(0.26–0.41)	3 (1.19%)	7 (0.37%)	7 (0.26%)	15 (0.25%)	50 (0.38%)	0.992	< 0.001	0.066
HPV-33	149	0.62	(0.52–0.72)	12 (4.76%)	20 (1.05%)	19 (0.70%)	42 (0.71%)	56 (0.43%)	37.537	< 0.001	− 0.313
HPV-35	119	0.50	(0.40–0.58)	9 (3.57%)	13 (0.68%)	22 (0.81%)	26 (0.44%)	49 (0.37%)	25.027	< 0.001	− 0.281
HPV-39	324	1.36	(1.21–1.50)	10 (3.97%)	44 (2.32%)	48 (1.76%)	69 (1.17)	153 (1.17%)	25.858	< 0.001	− 0.180
HPV-45	80	0.34	(0.26–0.40)	2 (0.79%)	10 (0.53%)	14 (0.51%)	13 (0.22%)	41 (0.31%)	4.187	0.041	− 0.131
HPV-51	273	1.14	(1.00–1.27)	14 (5.56%)	35 (1.84%)	37 (1.36%)	58 (0.98%)	129 (0.99%)	27.131	< 0.001	− 0.185
HPV-52	705	2.95	(2.73–3.16)	30 (11.90%)	69 (3.63%)	91 (3.34%)	151 (2.56%)	364 (2.78%)	22.886	< 0.001	− 0.099
HPV-53	399	1.67	(1.50–1.83)	11 (4.37%)	42 (2.21%)	54 (1.98%)	95 (1.61%)	197 (1.51%)	12.594	< 0.001	− 0.120
HPV-56	308	1.29	(1.14–1.43)	10 (3.97%)	31 (1.63%)	33 (1.21%)	73 (1.24%)	161 (1.23%)	4.866	0.027	− 0.069
HPV-58	495	2.07	(1.89–2.25)	22 (8.73%)	52 (2.74%)	65 (2.38%)	116 (1.96%)	240 (1.84%)	26.920	< 0.001	− 0.145
HPV-59	257	1.08	(0.94–1.20)	7 (2.78%)	42 (2.21%)	35 (1.28%)	46 (0.78%)	127 (0.97%)	21.993	< 0.001	− 0.167
HPV-66	209	0.88	(0.75–0.99)	11 (4.37%)	17 (0.89%)	27 (0.99%)	47 (0.80%)	107 (0.82%)	6.964	0.009	− 0.098
HPV-68	132	0.55	(0.45–0.64)	1 (0.40%)	6 (0.32%)	10 (0.37%)	32 (0.54%)	83 (0.63%)	5.044	0.025	0.171
HPV-82	92	0.39	(0.30-.46)	6 (2.38%)	16 (0.84%)	7 (0.26%)	18 (0.30%)	45 (0.34%)	12.653	< 0.001	− 0.184
HR + LR	707	2.96	(2.74–3.17)	42 (16.67%)	87 (4.58%)	85 (3.12%)	154 (2.61%)	339 (2.59%)	67.915	< 0.001	− 0.176
LR HPV	1001	4.19	(3.94–4.44)	27 (10.71%)	102 (5.37%)	127 (4.66%)	198 (3.35%)	547 (4.18%)	11.973	0.001	− 0.048
HPV-6	309	1.29	(1.15–1.43)	29 (11.51%)	44 (2.32%)	51 (1.87%)	52 (0.88%)	133 (1.02%)	91.034	< 0.001	− 0.294
HPV-11	194	0.81	(0.69–0.92)	18 (7.14%)	30 (1.58%)	25 (0.92%)	38 (0.64%)	83 (0.63%)	54.330	< 0.001	− 0.286
HPV-40	39	0.16	(0.11–0.21)	3 (1.19%)	6 (0.32%)	6 (0.22%)	10 (0.17%)	14 (0.11%)	12.752	< 0.001	− 0.351
HPV-42	84	0.35	(0.27–0.42)	0 (0.00%)	12 (0.63%)	9 (0.33%)	14 (0.24%)	49 (0.37%)	0.227	0.634	0.004
HPV-43	265	1.11	(0.97–1.24)	8 (3.17%)	27 (1.42%)	37 (1.36%)	68 (1.15%)	125 ().96%)	11.083	0.001	− 0.147
HPV-44	205	0.86	(0.74–0.97)	4	15	22	46	118	0.086	0.769	0.037
				(1.59%)	(0.79%)	(0.81%)	(0.78%)	(0.90%)			
HPV-55	209	0.88	(0.75–0.99)	12 (4.76%)	20 (1.05%)	38 (1.39%)	31 (0.52%)	108 (0.83%)	15.286	< 0.001	− 0.137
HPV-61	362	1.52	(1.36–1.67)	8 (3.17%)	30 (1.58%)	36 (1.32%)	75 (1.27%)	213 (1.63%)	0.072	0.789	0.047
HPV-81	285	1.19	(1.05–1.33)	8 (3.17%)	22 (1.16%)	37 (1.36)	71 (1.20%)	147 (1.12%)	2.584	0.108	− 0.066
HPV-83	48	0.20	(0.14–0.25)	1 (0.4%)	6 (0.32%)	6 (0.22%)	9 (0.15%)	26 (0.20%)	0.909	0.340	− 0.065

The five most common HR-HPV genotypes were HPV-52 (2.95%), HPV-16 (2.34%), HPV-58 (2.07%), HPV-53 (1.67%) and HPV-39 (1.36%). In addition, three most commonly detected LR-HPV genotypes were HPV-61 (1.52%), HPV-6 (1.29%) and HPV-81 (1.19%). We found significant differences for most genotypes from 2015 to 2019, except for HPV-26, HPV-42, HPV-44, HPV-61, HPV-81 and HPV-83 (*P* > 0.05; Table 2).

### Age-specific Prevalence of HPV infection

**Fig. 1:**
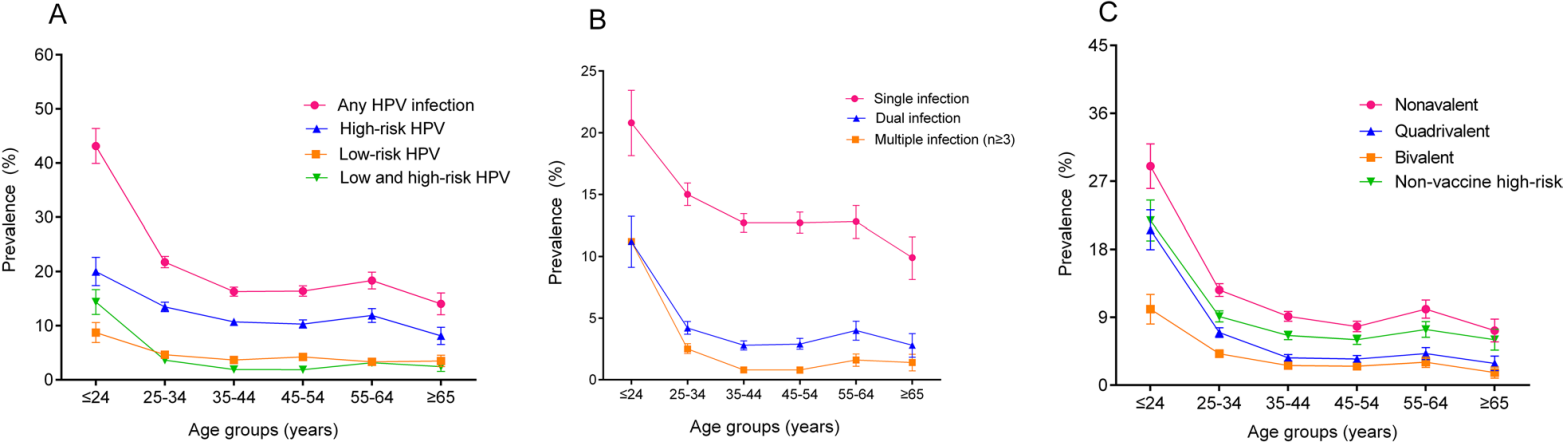
The prevalence of HPV infection by age groups. **A** any, high-risk, low-risk and low and high-risk HPV infection. **B** single, dual and multiple infection. **C** bivalent, quadrivalent, nonavalent vaccine targeting genotypes and non-vaccine high-risk genotypes. Error bars represent 95% confidence intervals

The age-specific prevalence of HPV infection is shown in Fig. 1. There were two peaks in the prevalence of overall HPV infection. The first peak found in women aged ≤ 24 years (43.14%), decreased sharply after the first peak, and maintained a plateau at middle age. The second peak observed at 55–64 years group (18.36%), followed by a moderately decline, and reached lowest prevalence at ≥ 65 years group (14.05%). HR-HPV infection also exhibited similar trend with the infection rates from 8.12% (≥ 65 years) to 20.02% (≤ 24 years). However, LR- and LR- and HR- mixed HPV infection curves showed relatively flat (Fig. 1A). Single HPV infection also peaked at ≤ 24 years, then dropped drastically with age, and stabilized in women aged 35–64 years without significant variation, and then decreased sharply again among ≥ 65 years group. Interestingly, the trend of the dual and multiple infection was similar to that overall HPV infection (Fig. 1B).

### Distribution of HPV genotypes

**Fig. 2:**
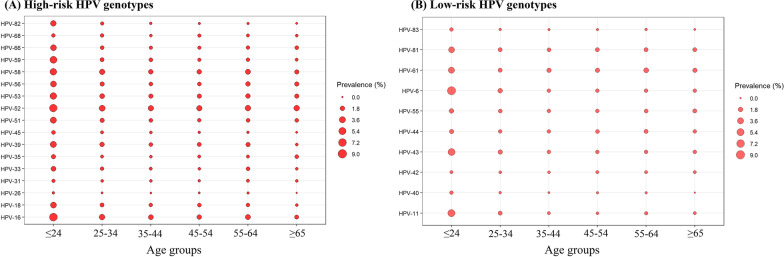
Bubble plots showing the relative prevalence of detected **A** 17 high-risk HPV genotypes and **B** 10 low-risk HPV genotypes across age groups

**Fig. 3:**
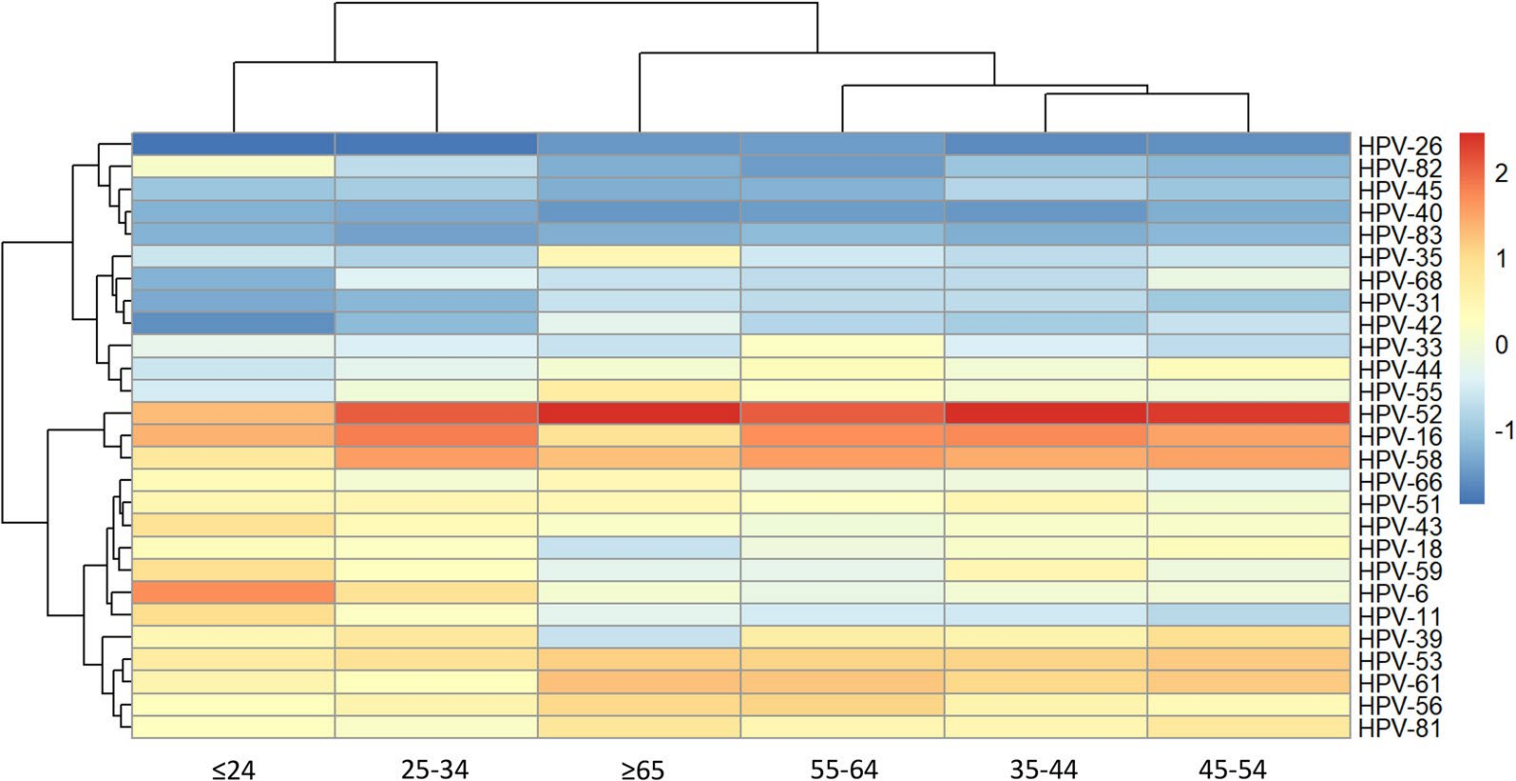
Heatmap of distribution of HPV genotypes in age groups. The prevalence was log-transformed to reduce the skewness of data

The distribution of HR-HPV and LR-HPV genotypes in different age groups are shown in Fig. 2. In the younger group (≤ 24 years), three most prevalent HR-HPV genotypes were HPV-16, HPV-52 and HPV-59, and the most prominent LR-HPV genotypes were HPV-6, HPV-11 and HPV-43. However, in the older group (≥ 55 years), HPV-52, HPV-16, HPV-58 and HPV-61, HPV-81, HPV-55 were the common HR-HPV and LR-HPV genotype, respectively. There was inconsistent distribution of HPV infection parttern among age groups. The highest percentage of single infection was detected in 35–44 years (77.9% in all HPV positive women). While, both dual and multiple infections were more frequently found in ≤ 24 years group (25.9%; Additional file 1: Table S1). Hierarchical clustering analysis showed that relative young-age group (≤ 34 years) and middle/older group (35–64 years and ≥ 65 years) shared similar distribution of HPV types (Fig. 3).

### Prevalence of HPV according to vaccine types

The prevalence of detected genotypes targeted by 2v, 4v, and 9v vaccines were 3.25%, 5.08%, and 10.42%, respectively, whereas the prevalence of non-vaccine HR-HPV genotypes was 7.70% as shown in Table 1. The prevalence of HPV genotypes grouped by age was shown in Fig. 1C. Moreover, we also compared the prevalence of patients with type included in 4v and 9v vaccines among age groups. A significant relationship was observed between vaccines (4v and 9v) and groups (*P* < 0.001), and a significantly higher coverage in 9v vaccine than in 4v vaccine for each age group (*P* < 0.001, Additional file 1: Table S2).

### Characteristics of HPV infection by geographical regions

**Fig. 4:**
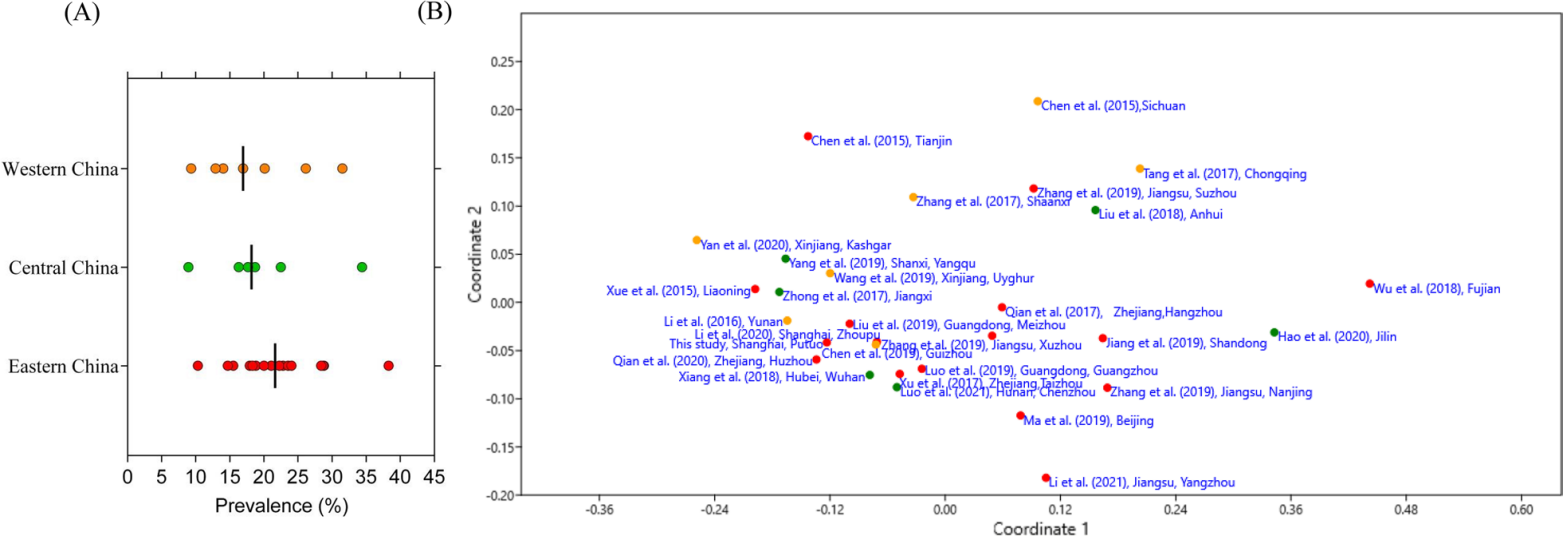
Prevalence and distribution of HPV by using both this study and collected data. **A** Prevalence of HPV stratified by geographical areas. Solid black lines represent the median value for each geographical group. **B** Non-metric Multi-Dimensional Scaling (NMDS) of HR-HPV genotypes using the Euclidean similarity index

We sorted out the reports according to geographical regions: eastern, central and western China based on previous published studies (Additional file 1: Table S3). The prevalence of those studies and NMDS plots of 17 HR-HPV are shown in Fig. 4. The median HPV prevalence in the eastern area of China (21.66%) was relatively higher than in the western (16.95%) and central (18.19%) regions. In general, the most commonly detected HR-HPV genotypes were HPV-16, HPV-52, HPV-58, HPV-53 and HPV-18.

## Discussion

### Comparison of the HPV prevalence in Shanghai with other regions of China

HPV prevalence rate varies greatly in different regions of China as it is influenced by multiple factors such as economic levels, living habits, awareness of prevention and screening, and HPV detection method sensitivity. In the current study, we found the overall prevalence of HPV infection was 18.81%, which was consistent with that reported in Hunan (18.71%) [16] and Guangdong (18.34%) [17], lower than in Chongqing (26.15%) [18], Jiangsu (26.92%) [19], Jilin (34.40%) [20] and Fujian (38.3%) [21], but higher than in Liaoning (10.30%) [22], Yunnan (12.90%) [23], Xinjiang (9.34–14.02%) [24, 25] and Huzhou (15.50%) [26]. A recent meta-analysis results showed that the overall prevalence of HR-HPV in mainland Chinese women was 19.0% [7]. HR-HPV prevalence rate in our study was 11.65%, which was 2.78 times the prevalence for LR-HPV infection and consistent with a recent national investigation in 2017 (12.1%) [27], lower than that in Jiangxi (19.53%) [28] and Shandong (24.2%) [29]. It is generally believed that the oncogenic HPV infection rate in developed economic areas was lower than in relatively underdeveloped areas [30]. The feasible reason for the lower HR-HPV prevalence rate in our study may be also associated with relatively developed economy and people,s advanced and better health awareness in Shanghai.

### Higher prevalence of HPV in younger women

Many studies have shown that HPV infection were significantly age-specific [31–34]. In the present study, overall HPV infection rate among young women (≤ 24 years, 43.14%) was much higher than that of other age groups, then the rate of HPV infection reduced sharply, which may be associated with their sexual behavior and attitude. However, decreasing trend stopped and rised slowly at 35–55 years, and slightly increased at 55–64 years (18.36%). Notably, the HR-HPV infection demonstrated the similar pattern. A previous study have been showed that the HR-HPV infection rate of Chinese women demonstrated “two-peak” pattern. The first peak presented at youngest age group (15–19 years), and the second peak observed at 50–60 years group [35]. In this study, the highest HR-HPV infection rate was also observed in the youngest age group (≤ 24 years), and followed by a less obvious peak for the 55–64 years group. While LR-HPV infection did not have the similar distribution. Compared with LR-HPV infection, HR-HPV infection was more likely to be prevalent, persistent and less likely to be cleared [36]. We also presented age-specific prevalence of single, dual and multiple HPV infections. The single genotype infection in aged ≤ 24 years group was higher than that of dual and multiple infections in other age groups, which also observed in northern Henan province of China [37]. Young women are thought to have more frequent sexual activities, more than one partner and relatively inadequate immune response, which makes them have a higher probability of exposure to HPV infection. For menopausal women, immune dysregulation would lead them unable effectively remove and inhibit the virus, may account for viral persistence or reactivation of latent HPV [38]. Therefore, further promotion of vaccination program and preventative screening strategies against cervical cancer for young women susceptible to HPV infection is necessary and urgent for this region. Additionally, cervical cancer screening program like HPV genotyping test is also valuable for perimenopausal women (≥ 55 years).

### High frequency of non-vaccine HR-HPV genotypes 53, 39, 56, 51 and 59 in Shanghai women

Knowledge of the genotype distribution of HPV in specific areas will enable the improvement of optimal protective strategies. Previous studies have indicated that HPV-52, -16 and -58 were the most common HPV genotype in many regional of China [16, 23, 39, 40]. In our study, the most common genotype was HPV-52, followed by HPV-16, -58, -53 and -39, which was consistent with the result in Guizhou [41]. HPV-52, -58, -16, -51 and -39 were the five most common HR-HPV genotypes in Yangzhou [42], and HPV-52, -16, -58, -39 and -51 in Wuhan [43]. It has been reported that HPV-52 and -58 were the more prevalent genotypes in Asia, especially in China, and infection with them may have association with the cervical cancer development [38, 44]. HPV-16 and HPV-18 were the most commonly encountered genotype worldwide, accounting for up to 70% of cervical cancers [3]. In our study, HPV-16 ranked second, whereas HPV-18 was only 9th common HR-HPV genotype. In Hangzhou, HPV-16 ranked first, and HPV-18 was 5th most common prevalent genotype [45], and 11th in Shanxi [46]. HPV-16 was the most common genotype coinfection with other types [27, 33]. HPV-18 was more common in other countries than in China [9]. Persistent infection with one or more high risk genotypes of HPV is one of the leading cause for cervical neoplasia [47]. It has been reported that HR-HPV genotypes can be found in more than 90% cervical cancer specimens [48]. HPV vaccination is an effective strategy for the primary prevention of HPV infection and the potential development of cervical neoplasia. Our study showed that bivalent, quadrivalent and nonavalent vaccines only covered 3.25%, 5.08%, and 10.42% HPV genotypes in this area. It was worth noting that in addition to HPV-52, -16 and -58, there was a high prevalence of HPV-53, -39, -56, -51 and -59 in this region. These HR-HPV genotypes are not included in current available vaccines, and may should be taken into account in the future HPV vaccines to reduce the risk of HPV-related cervical cancer development in Shanghai.

### Strengths and limitations of this study

This study provides a comprehensive analysis about the characteristics of HPV infection in Shanghai women. There are several limitations of this study. Firstly, this study was a only hospital-based survey including women who visited our hospital and received HPV DNA genotyping from Janauary 2015 to December 2019, which may not represent the general population in Shanghai. Secondly, there is no data of cervical lesion classification due to the inability to obtain complete cytological data of enrolled women. Thirdly, lack information of socieconomic status and sexual behaviors make it difficult to provide practical guidance in prevention of HPV infection.

## Conclusions

Our data disclosed a very high prevalence of HPV infection in younger women, suggesting the great necessary of HPV screening and vaccination among younger women. High frequency of non-vaccine-coverd HR-HPV genotypes demands a local epidemiological data-based new HPV vaccine in the future.

## Supplementary Information

**Additional file 1. **Prevalence of cervicovaginal human papillomaviruses  stratified by age and geographical areas.

## Data Availability

The original data that support the findings of this study are available upon reasonable request.

## Abbreviations

HPV: Human papillomavirus
HR: High-risk
LR: Low-risk
CFDA: China food and drug administration
95% CI: 95% Confidence interval
2v: Bivalent
4v: Quadrivalent
9v: Nonavalent
NMDS: Non-metric multi-dimensional scaling
