# Supplementary material for: Prevalence of cervicovaginal human papillomavirus infection and genotype distribution in Shanghai, China
Source: Virol J. 2022 Sep 12;19:146. doi: 10.1186/s12985-022-01879-y (PMC9465878; doi:10.1186/s12985-022-01879-y)
Supplement: Supplementary file 1 — Additional file 1. Prevalence of cervicovaginal human papillomaviruses stratified by age and geographical areas. [file 12985_2022_1879_MOESM1_ESM.docx]

**Prevalence of cervicovaginal human papillomavirus infection and genotype distribution in Shanghai, China**

Xiaoxiao Li^1^, Fenfen Xiang^1^, Junhua Dai^1^, Tao Zhang^1^, Zixi Chen^1^, Mengzhe Zhang^1^, Rong Wu^1^*, Xiangdong Kang^1^*

^1^Laboratory Medicine Department, Putuo Hospital, Shanghai University of Traditional Chinese Medicine, Shanghai, China

***Corresponding authors:** Rong Wu and Xiangdong Kang, Department of Laboratory Medicine, Putuo Hospital, Shanghai University of Traditional Chinese Medicine, Shanghai, China.

164 Lanxi Road, Shanghai 200062, P. R. China

E-mail: rong701@126.com (R. W.) and xd_kang@163.com (X. K.) ; Telephone: 021-51322043

**Table S1.** Percentage of HPV infection pattern by age group

|  | Age group | | | | | |
| --- | --- | --- | --- | --- | --- | --- |
|  | ≤24 | 25-34 | 35-44 | 45-54 | 55-64 | ≥65 |
| Infection pattern |  |  |  |  |  |  |
| Single infection | 48.2% | 69.0% | 77.9% | 77.6% | 69.6% | 70.2% |
| Dual infection | 25.9% | 19.4% | 17.1% | 17.8% | 21.7% | 19.9% |
| Multiple infection (n≥3) | 25.9% | 11.6% | 5.0% | 4.7% | 8.7% | 9.9% |

**Table S2.** Comparison of percentages of patients with genotypes covered by quadrivalent and nonavalent vaccine

| Group | Patients with genotype covered by 4v vaccine | Patients with genotype covered by 9v vaccine | McNemar test (4V vs.9v) *P*-value |
| --- | --- | --- | --- |
| ≤24 | 186 (20.58%) | 262 (28.98%) | < 0.001 |
| 25-34 | 415 (6.97%) | 751 (12.61%) | < 0.001 |
| 35-44 | 273 (3.63%) | 687 (9.12%) | < 0.001 |
| 45-54 | 204 (3.46%) | 458 (7.77%) | < 0.001 |
| ≥55 | 135 (3.76%) | 329 (9.17%) | < 0.001 |
| Multiple comparison χ^2^ test among groups | < 0.001 | < 0.001 | / |
| Total group | 1213 (5.08%) | 2487 (10.42%) | < 0.001 |

Table S3. Prevalence of overall and 17 high-risk HPV genotypes of a normal population in some areas of China

| Area  /city | No. of sample | Overall  positivity | HPV  -16 | HPV  -18 | HPV  -26 | HPV  -31 | HPV  -33 | HPV  -35 | HPV  -39 | HPV  -45 | HPV  -51 | HPV  -52 | HPV  -53 | HPV  -56 | HPV  -58 | HPV  -59 | HPV  -66 | HPV  -68 | HPV  -82 | Reference |
| --- | --- | --- | --- | --- | --- | --- | --- | --- | --- | --- | --- | --- | --- | --- | --- | --- | --- | --- | --- | --- |
| Shanghai, Putuo | 23866 | 18.81 | 2.34 | 1.00 | 0.05 | 0.34 | 0.62 | 0.50 | 1.36 | 0.34 | 1.14 | 2.95 | 1.67 | 1.29 | 2.07 | 1.08 | 0.88 | 0.55 | 0.39 | This study |
| Shanghai, Zhoupu | 59541 | 17.92 | 2.85 | 1.01 | NA | 0.97 | 1.28 | 0.29 | 1.46 | 0.29 | 1.45 | 3.58 | 1.81 | 0.57 | 2.64 | 0.40 | 0.83 | 0.99 | NA | Li *et al*. (2020) |
| Guangdong,  Meizhou | 36871 | 18.34 | 2.95 | 1.07 | 0.04 | 0.37 | 0.75 | 0.36 | 1.18 | 0.25 | 1.01 | 3.33 | 1.62 | 0.88 | 2.09 | 0.71 | 0.56 | 0.37 | 0.30 | Liu *et al*. (2019) |
| Guangdong,  Guangzhou | 211962 | 20.00 | 3.36 | 1.20 | NA | 0.72 | 1.05 | 0.20 | 1.40 | 0.29 | 1.10 | 5.12 | 1.91 | 0.61 | 2.70 | 0.49 | 0.85 | 1.26 | NA | Luo *et al*. (2019) |
| Zhejiang,  Taizhou | 37967 | 22.80 | 2.72 | 1.50 | 0.04 | 0.68 | 1.07 | 0.48 | 1.64 | 0.36 | 1.14 | 4.49 | 1.78 | 1.28 | 2.63 | 1.01 | 0.85 | 0.98 | 0.52 | Xu *et al*. (2017) |
| Zhejiang,  Hangzhou | 43804 | 22.26 | 4.81 | 1.90 | NA | 1.39 | 1.67 | 0.21 | 1.75 | 0.28 | 1.09 | 4.69 | 2.27 | 0.62 | 3.91 | 0.45 | 0.85 | 1.05 | NA | Qian *et al*. (2017) |
| Zhejiang,  Huzhou | 11506 | 15.50 | 1.91 | 0.94 | NA | 0.34 | 0.93 | 0.36 | 1.15 | 0.19 | 1.05 | 3.34 | 1.45 | 0.98 | 1.70 | 0.91 | 0.97 | 0.90 | 0.17 | Zhu *et al*. (2021) |
| Jiangsu,  Yangzhou | 34087 | 23.56 | 3.79 | 1.65 | NA | 1.12 | 1.05 | 0.95 | 2.39 | 0.48 | 2.77 | 6.15 | NA | 2.02 | 3.95 | 1.27 | 1.86 | 1.91 | 0.62 | Li *et al*. (2021) |
| Jiangsu,  Xuzhou | 26262 | 24.04 | 4.64 | 1.41 | NA | 0.89 | 1.35 | 0.83 | 0.70 | 0.37 | 2.05 | 4.69 | 2.30 | 1.51 | 2.57 | 1.17 | 1.22 | 2.00 | 0.13 | Zhang *et al*. (2019) |
| Jiangsu,  Nanjing | 26629 | 28.79 | 5.02 | 1.57 | NA | 1.31 | 1.75 | 0.92 | 1.21 | 0.51 | 2.44 | 6.21 | 3.24 | 2.08 | 3.58 | 1.33 | 1.31 | 2.11 | 0.21 | Zhang *et al*. (2019) |
| Jiangsu,  Suzhou | 9426 | 28.72 | 6.39 | 2.06 | NA | 1.51 | 2.64 | 1.38 | 0.90 | 0.62 | 1.06 | 3.01 | 0.66 | 1.77 | 3.50 | 1.10 | 0.98 | 1.80 | 0.48 | Zhang *et al*. (2019) |
| Beijing | 21239 | 21.06 | 4.44 | 1.47 | NA | 1.24 | 0.73 | 0.97 | 2.31 | 0.40 | 2.19 | 4.64 | NA | 2.05 | 4.28 | 1.49 | NA | 1.21 | NA | Ma *et al*. (2019) |
| Hunan,  Chenzhou | 214715 | 18.71 | 2.96 | 1.00 | NA | 0.70 | 1.05 | 0.21 | 1.43 | 0.22 | 1.08 | 5.12 | 1.84 | 0.48 | 2.51 | 0.37 | 0.58 | 1.06 | NA | Luo *et al*. (2021) |
| Xinjiang,  Uyghur | 37722 | 14.02 | 3.79 | 0.65 | 0.03 | 0.72 | 0.66 | 0.30 | 0.69 | 0.18 | 0.61 | 2.47 | 1.35 | 0.50 | 1.76 | 0.48 | 0.65 | 0.50 | 0.03 | Wang *et al*. (2019) |
| Xinjiang,  Kashgar | 12165 | 9.34 | 2.83 | 0.26 | NA | 0.99 | 0.13 | 0.25 | 0.49 | 0.30 | 0.51 | 0.70 | 0.14 | 0.24 | 0.40 | 0.40 | 0.48 | 0.88 | 0.12 | Yan *et al*. (2020) |
| Yunnan | 28457 | 12.90 | 2.32 | 0.84 | 0.04 | 0.55 | 0.62 | 0.22 | 0.83 | 0.21 | 0.51 | 2.81 | 0.56 | 0.77 | 1.48 | 0.53 | 0.33 | 0.70 | 0.38 | Li *et al*. (2016) |
| Shandong | 94489 | 28.40 | 5.80 | 1.70 | NA | 1.40 | 1.50 | 1.10 | 1.40 | 0.50 | 2.60 | 5.10 | NA | 2.30 | 3.50 | 1.60 | 1.80 | 2.10 | 0.20 | Jiang *et al*. (2019) |
| Chongqing | 40311 | 26.15 | 7.82 | 2.64 | 0.00 | 0.94 | 1.97 | 0.14 | 0.89 | 0.34 | 0.24 | 4.86 | 2.22 | 0.58 | 4.47 | 0.72 | 0.00 | 1.17 | 0.06 | Tang *et al*. (2017) |
| Jilin | 20648 | 34.40 | 7.80 | 2.40 | NA | 2.20 | 1.92 | 0.76 | 2.81 | 0.73 | 3.31 | 5.80 | 3.40 | 1.93 | 5.00 | 1.45 | 2.00 | 1.75 | NA | Hao *et al*. (2020) |
| Shanxi,  Yangqu | 10086 | 8.92 | 3.42 | 0.38 | NA | 0.52 | 0.58 | 0.30 | 0.35 | 0.05 | 0.60 | 1.77 | 0.76 | 0.72 | 1.47 | 0.29 | 0.73 | 0.52 | NA | Yang *et al*. (2019) |
| Jiangxi | 71435 | 22.49 | 2.60 | 0.64 | NA | 0.40 | 0.74 | 0.19 | 0.33 | 0.10 | 0.69 | 2.23 | 0.64 | 0.40 | 1.76 | 0.23 | 0.36 | 0.47 | NA | Zhong *et al*. (2017) |
| Anhui | 19753 | 16.30 | 7.79 | 0.90 | NA | 0.86 | 1.46 | 0.76 | 0.91 | 0.21 | 1.34 | 4.25 | 1.79 | 1.60 | 3.22 | 0.44 | 1.98 | 1.20 | NA | Liu *et al*. (2018) |
| Hubei, Wuhan | 13775 | 17.68 | 2.56 | 1.02 | NA | 0.52 | 1.02 | 0.11 | 1.71 | 0.35 | 1.68 | 4.23 | 1.65 | 0.43 | 2.37 | 0.48 | 0.55 | 1.13 | NA | Xiang *et al*. (2018) |
| Shaanxi | 38408 | 20.11 | 5.55 | 1.79 | 0.08 | 0.31 | 0.72 | 0.45 | 1.25 | 0.19 | 1.09 | 1.91 | 1.56 | 1.54 | 2.86 | 0.99 | 0.74 | 0.04 | 0.35 | Zhang *et al*. (2017) |
| Fujian | 8678 | 38.30 | 8.50 | 2.50 | NA | 2.00 | 2.60 | 0.80 | 2.10 | 0.50 | 1.70 | 7.90 | 3.50 | 1.50 | 6.20 | 1.10 | 2.00 | 2.00 | NA | Wu *et al*. (2018) |
| Liaoning | 6479 | 10.30 | 2.48 | 0.71 | NA | 0.62 | 0.72 | 0.14 | 0.35 | 0.14 | 0.09 | 1.84 | 1.26 | 0.17 | 1.31 | 0.23 | 0.48 | 0.60 | NA | Xue *et al*. (2015) |
| Tianjin | 2000 | 14.71 | 5.36 | 1.57 | NA | 0.66 | NA | 0.05 | 0.56 | 0.25 | 0.05 | 0.66 | 0.10 | 0.40 | 2.22 | 0.35 | 0.86 | 0.40 | NA | Chen *et al*. (2015) |
| Guizhou | 56768 | 16.95 | 3.02 | 0.94 | NA | 0.67 | 0.92 | 0.18 | 1.63 | 0.19 | 1.27 | 3.89 | 1.74 | 0.42 | 2.53 | 0.37 | 0.54 | 0.80 | NA | Chen *et al*. (2019) |
| Sichuan | 10682 | 31.50 | 7.40 | 1.88 | NA | 1.15 | 2.56 | 0.59 | 0.28 | 0.32 | 0.67 | 2.28 | 0.54 | 1.79 | 4.46 | 0.87 | 1.25 | 0.73 | NA | Chen *et al*. (2015) |
